# Mixture Random-Effect IRT Models for Controlling Extreme Response Style on Rating Scales

**DOI:** 10.3389/fpsyg.2016.01706

**Published:** 2016-11-02

**Authors:** Hung-Yu Huang

**Affiliations:** Department of Psychology and Counseling, University of TaipeiTaipei, Taiwan

**Keywords:** item response theory, extreme response style, mixture IRT models, Bayesian estimation, latent class

## Abstract

Respondents are often requested to provide a response to Likert-type or rating-scale items during the assessment of attitude, interest, and personality to measure a variety of latent traits. Extreme response style (ERS), which is defined as a consistent and systematic tendency of a person to locate on a limited number of available rating-scale options, may distort the test validity. Several latent trait models have been proposed to address ERS, but all these models have limitations. Mixture random-effect item response theory (IRT) models for ERS are developed in this study to simultaneously identify the mixtures of latent classes from different ERS levels and detect the possible differential functioning items that result from different latent mixtures. The model parameters can be recovered fairly well in a series of simulations that use Bayesian estimation with the WinBUGS program. In addition, the model parameters in the developed models can be used to identify items that are likely to elicit ERS. The results show that a long test and large sample can improve the parameter estimation process; the precision of the parameter estimates increases with the number of response options, and the model parameter estimation outperforms the person parameter estimation. Ignoring the mixtures and ERS results in substantial rank-order changes in the target latent trait and a reduced classification accuracy of the response styles. An empirical survey of emotional intelligence in college students is presented to demonstrate the applications and implications of the new models.

## Introduction

Likert-type scales and rating scales are widely used for self-report surveys in the social sciences and psychological assessments to measure a variety of latent traits, such as personality, interest, and attitude. Respondents are required to mark the degree of agreement or satisfaction on a number of ordered response categories in an inventory or questionnaire. The scale ratings by an individual may exhibit a consistent and systematic tendency to locate on a limited number of the available rating-scale options. Under these circumstances, we say that the individual exhibits a particular response style. Such styles can potentially distort the reliability and validity of investigative measures (De Jong et al., 2008; De Beuckelaer et al., 2010; Bolt and Newton, 2011; Plieninger and Meiser, 2014). Various response styles have been noted (Baumgartner and Steenkamp, 2001), which are assumed to be independent of the item content (Nunnally, 1978; Paulhus, 1991) and to be stable respondent characteristics over time (Berg, 1953; Hamilton, 1968; Bachman and O'Malley, 1984; Weijters et al., 2010).

Among these response styles, *extreme response style* (ERS), which is defined as a systematic tendency to select the end points of a rating scale, has attracted significant research attention (Hamilton, 1968; Greenleaf, 1992; Bolt and Johnson, 2009; Johnson and Bolt, 2010; Weijters et al., 2010; Bolt and Newton, 2011; Thissen-Roe and Thissen, 2013; Van Vaerenbergh and Thomas, 2013; Khorramdel and von Davier, 2014; Plieninger and Meiser, 2014; Jin and Wang, 2014a). Therefore, ERS and its opposite effect, mild response style (MRS), which is defined as a high frequency of endorsing middle responses of rating-scale items, are the focus of this study. We use the term “ERS tendency” to depict a respondent's tendency toward ERS or MRS because ERS and MRS cannot be observed at the same time on an item for the same respondent. Previous studies have indicated the relationships of ERS with demographic variables, psychological traits, and the features of the items (e.g., De Beuckelaer et al., 2010). The literature has indicated that the ERS tendency is independent of (or weakly correlated with) the substantive trait(s) and quite stable across contents (e.g., Weijters et al., 2010; Bolt and Newton, 2011; Thissen-Roe and Thissen, 2013; Jin and Wang, 2014a), which is adopted in this study. On the other hand, some researchers have concluded that longer items and ambiguity would elicit more extreme responses (Messick, 1991; Paulhus, 1991; Clarke, 2001; De Jong et al., 2008).

The existence of ERS interferes with test validity and inference and undermines cross-culture comparisons of scores (Hui and Triandis, 1989; De Jong et al., 2008; De Beuckelaer et al., 2010). In addition, the existence of ERS could result in biased estimates of item parameters and, in turn, could contaminate the precision of the target latent traits (Bolt and Newton, 2011; Jin and Wang, 2014a). For example, if high scores on a rating scale that measures the degree of depression often reflect ERS, the relationship between the self-report measure and other individual- or society-level variables (e.g., socioeconomic status; SES) would be biased because of the negative association between the SES level and ERS (Bolt and Newton, 2011).

Ignoring the possibility of extreme responses can spuriously violate measurement invariance or result in differential item functioning (DIF). In turn, these problems can confound the nuisance ERS effect and the measure-intended dimension (Cheung and Rensvold, 2000; Bolt and Johnson, 2009; Thissen-Roe and Thissen, 2013). In fact, if controls for ERS are absent, inferences regarding cross-cultural comparisons and measurement equivalence are misleading (Morren et al., 2012). Under real testing situations, differences in ERS tendencies among respondents and differences in item parameters among response styles may occur simultaneously; thus, their effects should be distinguished in a latent trait model. Unfortunately, no model that can address these issues has been developed yet. Therefore, this study attempts to develop a new type of latent trait model to simultaneously consider response styles and measurement invariance.

Respondents with similar response patterns can be grouped into a latent class by using mixture item response theory (IRT) models, in which different latent classes are allowed to have their specific set of item parameters such that a latent DIF analysis can be conducted (Cohen and Bolt, 2005; Cho and Cohen, 2010). Because different response styles may have different response patterns, mixture IRT models can be used to classify respondents with respect to their response styles. Mixture IRT models have been applied to analyze ERS data (e.g., Moors, 2008; Morren et al., 2012); however, existing models show some limitations in measuring the ERS degree for respondents and lack a justification for the assumption of zero variability within latent classes with respect to the ERS levels (see below for a further description). Thus, a new mixture IRT model for ERS is required to simultaneously enable the classification and quantification of respondents with respect to the ERS tendency, evaluate latent DIF items that are caused by the ERS effect, and model the effects of item characteristics that may elicit ERS on item responses as a function of the measure-intended latent trait. These requirements serve as the principal justification of model extension and are the most significant contribution of this study to the field of practical testing.

We build on the contributions of Jin and Wang (2014a) and extend their model by accommodating a random-effect variable to quantify the ERS tendencies and mixtures of latent classes that have homogeneous response patterns with respect to response styles. The random effect that is caused by ERS can be referred to as a specific latent trait that is not part of the intended construct and will be termed the “ERS dimension” in the remainder of this paper. Jin and Wang's model was used for three reasons: flexible extensions are feasible, the ERS tendency can be separated from the target trait and is easy to quantify, and the evaluation of extreme responses is not arbitrarily conducted by researchers (as is the case in nominal response models; see below for a detailed discussion).

This paper is structured as follows. We review the literature on the measurement of ERS. Next, we develop new mixture IRT models for ERS, and a series of simulation studies are conducted to evaluate the parameter recovery of the new class of models. Following these simulations, an empirical study is presented to demonstrate the applications and implications of the new models. We close this article by drawing conclusions regarding the new models and providing suggestions for future research.

### Measuring the ERS tendencies of respondents

A variety of model-based and non-model-based procedures have been provided to ameliorate and control for the effects of ERS in rating data. More simplistic approaches include frequency accounts of endpoint responses or the computation of the standard deviation of item scores within a respondent (Baumgartner and Steenkamp, 2001; Buckley, 2009). Such approaches, however, usually use homogeneous items (i.e., the same items are used to measure the substantive trait and ERS tendencies) and fail to explain the influence of the substantive trait (Bolt and Newton, 2011; Khorramdel and von Davier, 2014). In contrast, content heterogeneous items (i.e., a set of items that are administered for the sole purpose of identifying ERS) are used as an alternative to separate the effects of the target latent trait and ERS (Greenleaf, 1992), but the inclusion of content-uncorrelated items can undermine the measurement's validity (Bolt and Newton, 2011). Ipsative measures or normalizing item scores within subjects, in addition to the aforementioned simple strategies, have been developed but were found to eliminate construct differences along with response styles (Cheung and Rensvold, 2000; Thissen-Roe and Thissen, 2013).

In terms of model-based strategies for the investigation of ERS, IRT has been applied to analyze rating data to simultaneously separate item characteristics and person measures and to explain their interaction with ERS (De Jong et al., 2008; Bolt and Johnson, 2009). At least four variations of IRT models for ERS deserve further description. When a respondent responds to a rating item, the cognitive operation can be decomposed into multiple processes, and each cognitive process can be represented by a specific IRT model. Sequential response processes or a multinomial processing tree constitute tree-based IRT models, and the principal advantage of such models is that response-style-related and content-related processes are separable and the target latent traits and tendency of individuals toward ERS can be distinguished (Böckenholt, 2012; De Boeck and Partchev, 2012; Thissen-Roe and Thissen, 2013; Khorramdel and von Davier, 2014).

In addition to the attempted decomposition of response decisions, the multidimensional nominal response model (MNRM) has been developed to investigate ERS and other response styles by specifying category slope parameters for a random-effect variable to represent the tendency toward a particular type of response style (Bolt and Johnson, 2009; Johnson and Bolt, 2010; Bolt and Newton, 2011). Although, the MNRM allows individual differences in the target latent traits and ERS tendency, the nominal nature of the response options is not applicable to rating-scale items, extreme responses are determined arbitrarily, and two types of random effects (i.e., the target latent traits and ERS tendency) compensate each other without theoretical justification (see Thissen-Roe and Thissen, 2013; Jin and Wang, 2014a).

Invariance in the functioning of items across respondents is assumed in a typical ordinal IRT model. In some instances, respondents may use the rating scale in an expanded or contracted manner. Specifically, the proportional threshold model (Rossi et al., 2001) portrays ERS with regard to tendencies to select the extreme end options by allowing the threshold parameters to be proportional across individuals, in which a smaller distance between adjacent thresholds that an individual possesses is treated as a higher tendency to exhibit ERS (also see Böckenholt, 2001; Johnson, 2003). The response function in the proportional threshold model was constrained to the graded response model (Samejima, 1969), and the assumption of a symmetric vector of thresholds was not practical under real testing situations. Recently, Jin and Wang (2014a) proposed a class of IRT models for ERS measures where a variety of ordered-response categorical IRT models were extended to quantify the ERS degree for respondents by treating thresholds as random effects rather than fixed effects.

Another approach to address the effects of ERS on person measures adopts mixture IRT models to represent different levels of ERS between respondents in the form of latent classes (Rost et al., 1997; von Davier et al., 2007; Moors, 2008; Van Rosmalen et al., 2010). In these authors' modeling frameworks, a latent class that has a small distance between adjacent thresholds is identified as having a high likelihood of exhibiting ERS. Although, mixture IRT approaches can detect different latent classes relative to different levels of extreme responses in an exploratory way, adjusting latent trait estimates in response to ERS and quantifying the ERS tendency at the individual level are difficult (Austin et al., 2006; Bolt and Newton, 2011).

Morren et al. (2012) extended the MNRM for ERS (e.g., Bolt and Newton, 2011) using latent classes to identify three types of response styles (normal, ERS, and MRS) and allowed different latent means with respect to the ERS tendency in the three latent classes. This model attempted to combine a nominal response model with a finite latent class model and quantify the ERS tendency. However, the assumption of no variability in the ERS dimension within each latent class limited its application because individual differences in the ERS tendency between respondents would be expected even in the same latent group. In addition, the use of such a mixture MNRM to measure the ERS tendency confronts the same problems as the MNRM.

In this study, previous ordered-category IRT models for ERS (Jin and Wang, 2014a) were extended by incorporating mixtures of latent classes that represent different response styles and a random effect that simultaneously controls for local item-dependence and quantifies the ERS tendency. This approach essentially combines the advantages of mixture IRT models and random-effect IRT models. This new approach to ERS will be described in detail in the following section.

### Mixture IRT models for ERS

Many polytomous IRT models have been proposed for ordered categorical responses that are commonly found in attitude and personality assessment to represent the non-linear relationship between the latent trait levels and the probability of responding to a particular category or option. For example, the generalized partial credit model (GPCM; Muraki, 1992) can be formulated, and the log-odds of receiving a score *j* over score *j* − 1 is expressed as

(1) $$\begin{array}{l} \log\left(\frac{P_{nij}}{P_{ni\left(j-1\right)}}\right)=\alpha_{i}\theta_{n}-\left(\beta_{i}+\tau_{ij}\right), \end{array}$$

where *P*_*nij*_ and *P*_*ni*(*j* − 1)_ are the probabilities of obtaining scores *j* and *j*-1 on item *i* for respondent *n*, respectively; θ_*n*_ is the level of the latent trait of respondent *n* and is assumed to follow a standard normal distribution; α_*i*_ is the discriminating power of item *i* with respect to the latent trait; β_*i*_ is the overall difficulty parameter of item *i*; and τ_*ij*_ is the *j*th threshold parameter for item *i*. This model assumes that a unidimensional latent trait underlies item responses and local item independence is satisfied (i.e., the items in the test are not related to each other when the trait level is controlled), but one case violates this assumption if response styles are observed.

Jin and Wang (2014a) incorporated a random-effect variable into the GPCM when considering the effect of ERSs on the probability function to quantify the tendency toward ERS, which is denoted as the ERS-GPCM and can be expressed as

(2) $$\begin{array}{l} \log\left(\frac{P_{nij}}{P_{ni\left(j-1\right)}}\right)=\alpha_{i}\theta_{n}-\left(\beta_{i}+\omega_{n}\tau_{ij}\right), \end{array}$$

where ω_*n*_ is a weight parameter for respondent *n* in the ERS dimension to control the distance between adjacent thresholds and is assumed to follow a lognormal distribution with a mean of zero and variance of $\sigma_{\omega }^{2}$; the other variables are the same as described above. A ω parameter that is lower than one can produce a contracted scale such that the extreme responses are more likely to be endorsed. In contrast, a ω parameter that is greater than one increases the likelihood of exhibiting a tendency toward MRS because the scale is expanded and the distance between thresholds becomes large. When ω = 1 for all respondents, the ERS-GPCM is equivalent to the GPCM, which indicates μ_ω_ = 0 and $\sigma_{\omega }^{2}=0$ in the ERS model.

This study develops a mixture ERS-IRT model to distinguish the effects of the ERS dimension and the intended-to-be-measured construct dimension on item functioning. In this model, the latent classes with respect to different response styles (i.e., three response styles: normal, ERS, and MRS) are considered and items that may function differently because of different response patterns among latent classes can be detected simultaneously. We thus extend the ERS-GPCM to

(3) $$\begin{array}{l} \log\left(\frac{P_{ngij}}{P_{ngi\left(j-1\right)}}\right)=\alpha_{i}\theta_{ng}-\left(\beta_{ig}+\omega_{ng}\tau_{ij}\right), \end{array}$$

(4) $$\theta_{ng}\sim \log N\left(\mu_{\theta_{\text{g}}},\;\sigma_{\theta_{\text{g}}}^{2}\right),$$

and

(5) $$\omega_{ng}\sim \log N\left(\mu_{\omega_{\text{g}}},\;\sigma_{\omega_{\text{g}}}^{2}\right),$$

where *P*_*ngij*_ and *P*_*ngi*(*j*−1)_ are the probabilities of obtaining scores *j* and *j*-1 on item *i* for respondent *n* from latent class *g*, respectively; θ_*ng*_ and ω_*ng*_ are the latent trait and weight parameters of respondent *n* within latent class *g*, respectively, and are assumed to be independent; β_*ig*_ is the overall difficulty parameter of item *i* for class *g*; and the other variables are the same as defined above. The discrimination parameters are assumed to be invariant across latent classes and do not have a subscript *g* because the difference in item location or item difficulty between classes is often of interest in mixture IRT literature (for a review, see Paek and Cho, 2015). Thus, this study can be compared to other studies. In addition, such a constraint implies that metric invariance is satisfied but scalar invariance is not (De Boeck et al., 2011). When appropriate, the assumption of equal discrimination parameters across latent classes can be easily relaxed and metric invariance can be evaluated.

The θ target latent trait is constrained to be normally distributed with a mean of zero and variance of one and the ω weight parameter is constrained to be one for all respondents to identify a mixture ERS-GPCM for a normal class without exhibiting a tendency toward ERS or MRS. In addition, the mean item overall difficulty is set to zero within each class to construct a common metric over the classes, as in the latent DIF analysis (Cho and Cohen, 2010). As in other latent class models, the phenomenon of label switching may occur. In this study, we impose an ordinal constraint on the mixing proportions to ensure that the normal class has a higher proportion than the other classes to avoid label switching (McLachlan and Peel, 2000).

The item's discriminating power (slope parameter) is indicative of a respondent's standing on the random-effect variables and is often treated as an indicator to represent the influence of the random effects on test items. Therefore, we can assume a distinct set of discrimination parameters for the ω random-effect weight parameter and can interpret the discriminating power of an individual item on the ω parameter as the extent to which that item is influenced by the ERS dimension. A well-designed item is expected to have a lower discrimination parameter on the ω dimension. The item characteristics and attributes are found to substantially influence the response styles, so extreme responses may be easily elicited when, for example, items are presented with more words or characters (Messick, 1991; Clarke, 2001; De Jong et al., 2008) or in an ambiguous manner (Messick, 1991; Paulhus, 1991; Johnson and Bolt, 2010; Bolt and Newton, 2011; Thissen-Roe and Thissen, 2013). A generalized mixture ERS-GPCM can be formulated by adding a different set of discrimination parameters (α_*i*2_) to the ω random effect in Equation (3):

(6) $$\begin{array}{l} \log\left(\frac{P_{ngij}}{P_{ngi\left(j-1\right)}}\right)=\alpha_{i1}\theta_{ng}-\left(\beta_{ig}+\alpha_{i2}\omega_{ng}\tau_{ij}\right). \end{array}$$

Unfortunately, this general mixture ERS-GPCM is not identifiable unless a further constraint is imposed because three types of parameters are multiplied mutually and no unique solution exists. In the framework of the bifactor model (Gibbons and Hedeker, 1992), the discriminating power of an item on the secondary dimension (i.e., ω) can be low if that item has high discriminating power on the intended-to-be-measured latent trait (Li et al., 2006). The relationship between an item's discrimination with respect to θ and ω can be formulated by imposing a constraint on the item's discriminating power on ω. A multidimensional discrimination parameter (MDP) is used to represent the inverse relationship between α_*i*1_ and α_*i*2_, which is assumed to be constant across items:

(7) $$\alpha_{i2}=\sqrt{MDP^{2}\;-\;\alpha_{i1}^{2}}.$$

The mixture ERS-GPCM with constraints on discrimination parameters (denoted as a mixture ERS-GPCM-CD) is an extension of the mixture ERS-GPCM that allows different items to have specific discriminating power on ω but assumes consistent discrimination across items, which apportions a different amount of discrimination to θ and ω. Li et al. (2006) argued that such a model is more realistic if the test is designed such that the discriminating power of the test items on the secondary dimension is small. As is the case with the mixture ERS-GPCM, the ω dimension is expected to have little effect on an item if that item is developed and written appropriately (e.g., appropriate word count or less ambiguous in the item stem).

Equation (6) is re-parameterized as follows to ensure that a lower discrimination parameter with respect to ω can reduce the effects of response styles on the item responses of a respondent with ERS or MRS tendencies (i.e., a ω value far from one) for rating-scale items:

(8) $$\begin{array}{l} \log\left(\frac{P_{ngij}}{P_{ngi\left(j-1\right)}}\right)=\alpha_{i1}\theta_{ng}-\left(\beta_{ig}+\eta_{ni}\tau_{ij}\right), \end{array}$$

and

(9) $$\begin{array}{l} \eta_{ni}=\exp[\alpha_{i2}\times \text{log}\left(\omega_{ng})\right], \end{array}$$

where ω is assumed to follow a lognormal distribution. Suppose three respondents with an ω value of 0.5 (ERS), 1 (normal), and 1.5 (MRS); given a response to an item with α_*i*2_ = 0.2, their corresponding η values are computed as 0.87, 1.00, and 1.08, respectively, which suggests that the response style effects substantially declined and the responses of the three respondents were more consistent with those from a typical GPCM function.

Multiple tests that measure multiple latent traits are commonly administered to individuals, and these tests can constitute a test battery under real testing situations. When a scale consists of multiple subscales and each subscale measures a specific latent trait, multidimensional IRT models should be adopted because they are more statistically efficient than unidimensional IRT models (Cheng et al., 2009). In our modeling framework, for example, the multidimensional approach of the mixture ERS-GPCM-CD can be expressed as

(10) $$\begin{array}{l} \log{\left(\frac{P_{ngij}}{P_{ngi\left(j-1\right)}}\right)}_{s}=\alpha_{i1s}{\;\theta }_{ngs}-\left(\beta_{igs}+\eta_{nis}\tau_{ijs}\right), \end{array}$$

and

(11) $$\begin{array}{l} \eta_{nis}=\exp[\sqrt{MDP_{\text{s}}^{2}\;-\;\alpha_{i1s}^{2}}\times \text{log}(\omega_{ngs})], \end{array}$$

where the subscript *s* denotes subscales. The θ variables are assumed to follow a multivariate normal distribution and to be independent of the other ω variables. Each person can be assumed to have a common ω value across subscales; thus, ω_*ngs*_ simplifies to ω_*ng*_ because the response styles are often assumed to be independent of the test contents. Similarly, a multidimensional mixture ERS-GPCM can be derived from the constraints of equal discrimination power on the ω random-effect variable.

In addition to the multidimensional approach, a mixture ERS-IRT model is very flexible for any model extension. For example, when a common set of threshold parameters are set to the item response function in the GPCM, the GPCM simplifies to the generalized rating scale model (GRSM) and its corresponding mixture ERS-IRT model can be formulated (denoted as a mixture ERS-GRSM). When an equal discrimination parameter is set for all the items, the partial credit model (PCM) arises and corresponds to a mixture ERS-PCM. The rating scale model (RSM) and its corresponding mixture ERS-IRT model in a mixture ERS-RSM can be formulated when the threshold and discrimination parameters are constrained simultaneously.

## Methods

### Simulation design

Two simulation studies were conducted to evaluate the quality of parameter estimation for a mixture ERS-GPCM and mixture ERS-GPCM-CD, each of which assumed that a single target latent trait underlay the data responses of the respondents. During the first simulation study, the mixture ERS-GPCM was used to generate the responses of the respondents, and three factors were manipulated, namely, (a) sample sizes of 1000 and 2000, (b) test lengths of 20 and 40 rating-scale items, and (c) numbers of item categories of 4 and 6, which were consistent with the study by Jin and Wang (2014a). Three latent classes that represented normal, ERS, and MRS results were generated, with each having a proportion of 50, 25, and 25%, respectively. For two types of random-effect variables, θ was randomly sampled as *N*(0, 1) for the three response style classes, and ω was set to one for the normal response class and was generated from lognormal(−1, 0.4^2^) and lognormal(1,0.4^2^) for the ERS class and MRS class, respectively. Consequently, ω was between 0.17 and 0.82 for the ERS class and between 1.22 and 2.72 for the MRS class within two standard deviations. Setting a dominant proportion for the normal class and low variation in ω for the ERS and MRS classes was reasonable because previous studies indicated that nearly one-third of individuals exhibited ERS (Jin and Wang, 2014a) and that the variation in the ERS effect was lower than the variation in the substantive trait (Bolt and Newton, 2011).

The item discrimination parameters were generated from lognormal(1,0.2^2^). The overall item difficulty parameters for the normal class were generated from a uniform distribution between −1.5 and 1.5, and the mean item difficulty was set to zero. A value of 0.5 or −0.5 was uniformly added to the item difficulties for the ERS and MRS classes; thus, the mean item difficulty in the test for the three classes was equal. The threshold parameters were set to −0.6, 0, and 0.6 for the 4-category items and −0.8, −0.4, 0, 0.4, and 0.8 for the 6-category items. The specifications of the model parameters were consistent with those that are commonly found in practice and similar to previous research (e.g., Li et al., 2006; Morren et al., 2012; Jin and Wang, 2014a; Huang, 2014, 2015, 2016). Each condition was replicated 30 times. Accordingly, a total of 240 (2 sample sizes × 2 test lengths × 2 category-response types × 30 replications) data sets were analyzed.

The second simulation study simulated data by using a mixture ERS-GPCM-CD, in which the manipulated factors and generated values for item and person parameters were the same as in the first simulation study. The generated discrimination parameters on θ were between 0.623 and 1.452, so the MDP was set to 1.50, and the generated discrimination parameters on ω were set between 0.048 and 0.877 to represent the influence of ω on the items, ranging from trivial to mild. Each condition had 30 replications, and the combination of manipulated factors and replications resulted in 240 experiments.

### Analysis

The proposed mixture ERS-IRT models are sophisticated and difficult to integrate over multiple random effects with likelihood-based estimation. Thus, Bayesian estimation with the Markov chain Monte Carlo (MCMC) method was used to calibrate the model parameters. The WinBUGS freeware program (Spiegelhalter et al., 2003) was applied to this study for data analysis because WinBUGS is flexible enough to enable users to specify their customized models and has been widely implemented in mixture IRT models (e.g., Cohen and Bolt, 2005; Cho and Cohen, 2010).

In Bayesian estimation, a statistical model and a set of prior distributions for model parameters are required to produce a joint posterior distribution, and MCMC methods are used to yield a specific posterior distribution for each parameter through sequential sampling. The prior distributions of model parameters were as follows, in accordance with previous studies (Cohen and Bolt, 2005; Li et al., 2006; Cho and Cohen, 2010; Huang et al., 2013; Jin and Wang, 2014a). We specified a normal prior distribution with a mean of zero and variance of four for all location and threshold parameters and a lognormal prior distribution with a mean of zero and variance of one for the slope parameters and the MDP. For the normal response class, θ and ω were constrained as their generated distributions for model identification. For the ERS and MRS classes, the mean and inverse variance of the normal distribution for θ were assumed to have a normal prior distribution with a mean of zero and variance of 10 and a gamma prior distribution with both hyper-parameters equaling 0.01. Finally, a categorical prior distribution was set for the indicators of the latent classes, and an ordinal constraint was imposed on the mixing proportions to ensure that the normal class had a higher proportion than the other classes.

In Bayesian mixture IRT model analyses, a label-switching problem may occur across iterations either within a single MCMC chain or between multiple MCMC chains. Several approaches have been developed to avoid the occurrence of label switching (Meyer, 2010). In this study, the author constrained the normal class to have a dominant proportion and assumed the mean of the lognormal distribution for ω to have a normal prior distribution with a mean of −0.5 and 0.5 for the ERS and MRS classes, respectively, and a variance of 10 for both the ERS and MRS classes. The prior distribution for the inverse variance of the ω lognormal distribution was set to be the same as in the setting of the θ normal distribution. The prior distributions for the ERS and MRS classes were less vague because a theoretical hypothesis for the ERS and MRS member characteristics was created in this study and because vague prior distributions have been found to result in technical WinBUGS problems in mixture IRT modeling (Frederickx et al., 2010). The multivariate potential scale reduction factor (Brooks and Gelman, 1998), which has three parallel chains for five randomly selected simulated datasets under each condition, was computed to assess the convergence of parameter estimation and to monitor whether the phenomenon of label switching occurred. We used 15,000 iterations, with the first 5000 iterations defined as the burn-in period because all the multivariate potential scale reduction factors were close to unity, and no label switching was observed. The WinBUGS commands for the proposed models are available upon request.

The bias and root mean square error (RMSE) were computed to assess the parameter recovery for each estimator:

(12) $$\begin{array}{l} \text{Bias}\left(EAP\left(\zeta \right)\right)=\overset{R}{\underset{r=1}{\sum }}\left(EAP\left(\zeta_{r}\right)-\zeta \right)/R, \end{array}$$

(13) $$\begin{array}{l} \text{RMSE}\left(EAP\left(\zeta \right)\right)=\sqrt{\overset{R}{\underset{r=1}{\sum }}{\left(EAP\left(\zeta_{r}\right)-\zeta \right)}^{2}/R}, \end{array}$$

where *R* equals the number of replications to evaluate the model parameter recovery and the number of individuals to assess the person parameter recovery; and ζ and *EAP*(ζ) are the generated value and the expected a posterior (EAP) estimate, respectively. Class membership recovery was also evaluated by computing the correct classification rates for each latent class. We predicted the results as follows: (a) the parameters could be recovered satisfactorily for both the mixture ERS-GPCM and mixture ERS-GPCM-CD; (b) the latent classes in which individuals exhibited a specific response style could be correctly identified; (c) a longer test and larger sample size would increase the precision of the parameter estimation; (d) the use of 6-point rating items would outperform the use of 4-point rating items with respect to parameter recovery; and (e) ignoring the mixtures and ERS by fitting a single-class ERS-GPCM and GPCM to the mixture ERS-GPCM data would result in poor estimations of the target latent trait.

## Results

### Parameter recovery for both simulations

The quality of parameter estimation in the simulations was assessed from the means and standard deviations of the bias and RMSE values because space constraints should be considered. Table 1 presents the parameter recovery when the mixture ERS-GPCM was used to generate data responses for 4-point rating items. The bias values were very close to zero under most conditions, with the exception of the ω mean estimate for a short test and small sample size. With respect to the RMSE values, a long test length and large sample size resulted in smaller RMSE values and yielded better parameter recovery. In addition, the location parameters in the normal class were estimated more precisely than the other two classes because the normal class had a dominant proportion in the sample size. The same results applied to the condition when item responses were generated with 6-point rating items, as shown in Table 2. In comparison, the parameter recovery of the 6-point rating items was better than that of 4-point rating items, as indicated by the smaller RMSE values in Table 2.

**Table 1:** **Mean bias and RMSE of the model parameter estimates for the mixture ERS-GPCM on 4-point items**.

**Sample size**	**1000**						**2000**					
**Response style**	**Normal**		**ERS**		**MRS**		**Normal**		**ERS**		**MRS**	
**Criterion**	**Bias**	**RMSE**	**Bias**	**RMSE**	**Bias**	**RMSE**	**Bias**	**RMSE**	**Bias**	**RMSE**	**Bias**	**RMSE**
**PARAMETER**												
**Test length = 20**												
Discrimination	0.022	0.102	—	—	—	—	0.011	0.072	—	—	—	—
Location	0.000	0.132	0.000	0.129	0.000	0.192	0.000	0.084	0.000	0.090	0.000	0.122
Threshold	0.000	0.148	—	—	—	—	0.000	0.092	—	—	—	—
$\bar{\theta }$	—	—	0.003	0.088	0.029	0.131	—	—	0.023	0.054	−0.036	0.077
Var(θ)	—	—	0.024	0.236	0.013	0.292	—	—	−0.027	0.190	0.004	0.218
$\bar{\omega }$	—	—	−0.241	0.333	−0.137	0.169	—	—	−0.136	0.207	−0.062	0.092
Var(ω)	—	—	0.032	0.127	0.001	0.049	—	—	0.002	0.109	−0.012	0.036
**Test length = 40**												
Discrimination	0.025	0.080	—	—	—	—	0.007	0.054	—	—	—	—
Location	0.000	0.079	0.000	0.099	0.000	0.142	0.000	0.053	0.000	0.071	0.000	0.097
Threshold	0.000	0.093	—	—	—	—	0.000	0.057	—	—	—	—
$\bar{\theta }$	—	—	0.003	0.097	0.008	0.087	—	—	0.007	0.054	0.002	0.051
Var(θ)	—	—	−0.012	0.128	−0.022	0.182	—	—	0.000	0.113	−0.025	0.124
$\bar{\omega }$	—	—	−0.102	0.221	−0.065	0.091	—	—	−0.051	0.129	−0.038	0.057
Var(ω)	—	—	−0.009	0.103	−0.004	0.023	—	—	−0.014	0.097	0.001	0.021

*—, not applicable because of model constraints*.

**Table 2:** **Mean bias and RMSE of the model parameter estimates for the mixture ERS-GPCM on 6-point items**.

**Sample size**	**1000**						**2000**					
**Response style**	**Normal**		**ERS**		**MRS**		**Normal**		**ERS**		**MRS**	
**Criterion**	**Bias**	**RMSE**	**Bias**	**RMSE**	**Bias**	**RMSE**	**Bias**	**RMSE**	**Bias**	**RMSE**	**Bias**	**RMSE**
**PARAMETER**												
**Test length = 20**												
Discrimination	0.033	0.084	—	—	—	—	0.010	0.057	—	—	—	—
Location	0.000	0.076	0.000	0.087	0.000	0.138	0.000	0.046	0.000	0.062	0.000	0.091
Threshold	0.000	0.127	—	—	—	—	0.000	0.079	—	—	—	—
$\bar{\theta }$	—	—	0.020	0.083	0.004	0.107	—	—	0.006	0.056	0.036	0.084
Var(θ)	—	—	−0.074	0.184	−0.099	0.212	—	—	0.014	0.139	−0.040	0.130
$\bar{\omega }$	—	—	−0.190	0.247	−0.115	0.131	—	—	−0.057	0.107	−0.057	0.073
Var(ω)	—	—	0.002	0.118	−0.015	0.031	—	—	−0.013	0.080	0.002	0.024
**Test length = 40**												
Discrimination	0.024	0.073	—	—	—	—	0.013	0.048	—	—	—	—
Location	0.000	0.068	0.000	0.076	0.000	0.122	0.000	0.045	0.000	0.054	0.000	0.086
Threshold	0.000	0.113	—	—	—	—	0.000	0.074	—	—	—	—
$\bar{\theta }$	—	—	−0.011	0.081	0.000	0.068	—	—	−0.008	0.057	−0.006	0.046
Var(θ)	—	—	−0.001	0.150	−0.065	0.122	—	—	−0.019	0.090	−0.050	0.111
$\bar{\omega }$	—	—	−0.110	0.152	−0.086	0.092	—	—	−0.051	0.080	−0.051	0.058
Var(ω)	—	—	0.015	0.098	−0.008	0.023	—	—	−0.017	0.065	−0.003	0.015

*—, not applicable because of model constraints*.

The results from when the mixture ERS-GPCM-CD was used to simulate data responses are summarized in Tables 3, 4 for the 4- and 6-point rating items, respectively. Similar results and patterns to those in the mixture ERS-GPCM can be found in the mixture ERS-GPCM-CD, and the same conclusions that were derived from the above simulation directly apply here. Differences in the parameter estimation between the mixture ERS-GPCM-CD and mixture ERS-GPCM were trivial, as indicated by their similar parameter recovery. In addition, the MDP parameter was estimated satisfactorily, which indicates that the approach of imposing a distinct set of discrimination parameters for the ω weight parameter was acceptable in the mixture ERS-IRT models. In summary, the parameters in both the mixture ERS-GPCM and the mixture ERS-GPCM-CD could be recovered well with longer tests, larger samples, and more response options, and the WinBUGS program with Bayesian estimation could provide precise model parameter estimates.

**Table 3:** **Mean bias and RMSE of the model parameter estimates for the mixture ERS-GPCM-CD on 4-point items**.

**Sample size**	**1000**						**2000**					
**Response style**	**Normal**		**ERS**		**MRS**		**Normal**		**ERS**		**MRS**	
**Criterion**	**Bias**	**RMSE**	**Bias**	**RMSE**	**Bias**	**RMSE**	**Bias**	**RMSE**	**Bias**	**RMSE**	**Bias**	**RMSE**
**PARAMETER**												
**Test length = 20**												
Discrimination	0.029	0.099	—	—	—	—	0.010	0.065	—	—	—	—
Location	0.000	0.128	0.000	0.132	0.000	0.194	0.000	0.069	0.000	0.088	0.000	0.129
Threshold	0.000	0.145	—	—	—	—	0.000	0.081	—	—	—	—
$\bar{\theta }$	—	—	0.005	0.081	0.032	0.132	—	—	0.003	0.054	−0.014	0.085
Var(θ)	—	—	−0.053	0.213	−0.099	0.212	—	—	−0.017	0.172	0.006	0.155
$\bar{\omega }$	—	—	−0.227	0.440	−0.125	0.199	—	—	−0.112	0.281	−0.033	0.101
Var(ω)	—	—	0.056	0.312	−0.024	0.061	—	—	0.034	0.175	−0.011	0.034
MDP	0.058	0.115	—	—	—	—	0.034	0.086	—	—	—	—
**Test length = 40**												
Discrimination	0.011	0.073	—	—	—	—	0.002	0.052	—	—	—	—
Location	0.000	0.076	0.000	0.099	0.000	0.146	0.000	0.054	0.000	0.073	0.000	0.100
Threshold	0.000	0.092	—	—	—	—	0.000	0.059	—	—	—	—
$\bar{\theta }$	—	—	−0.038	0.080	−0.021	0.078	—	—	−0.013	0.056	0.007	0.048
Var(θ)	—	—	0.026	0.156	−0.009	0.159	—	—	0.023	0.122	−0.008	0.113
$\bar{\omega }$	—	—	−0.050	0.168	−0.085	0.114	—	—	−0.023	0.138	−0.043	0.079
Var(ω)	—	—	−0.019	0.086	−0.020	0.032	—	—	−0.016	0.095	−0.003	0.017
MDP	0.057	0.091	—	—	—	—	0.016	0.052	—	—	—	—

*—, not applicable because of model constraints*.

**Table 4:** **Mean bias and RMSE of the model parameter estimates for the mixture ERS-GPCM-CD on 6-point items**.

**Sample size**	**1000**						**2000**					
**Response style**	**Normal**		**ERS**		**MRS**		**Normal**		**ERS**		**MRS**	
**Criterion**	**Bias**	**RMSE**	**Bias**	**RMSE**	**Bias**	**RMSE**	**Bias**	**RMSE**	**Bias**	**RMSE**	**Bias**	**RMSE**
**PARAMETER**												
**Test length = 20**												
Discrimination	0.019	0.079	—	—	—	—	0.004	0.051	—	—	—	—
Location	0.000	0.078	0.000	0.089	0.000	0.137	0.000	0.048	0.000	0.060	0.000	0.093
Threshold	0.000	0.126	—	—	—	—	0.000	0.079	—	—	—	—
$\bar{\theta }$	—	—	−0.002	0.079	0.027	0.088	—	—	−0.024	0.062	−0.014	0.075
Var(θ)	—	—	−0.054	0.200	−0.050	0.192	—	—	−0.016	0.140	−0.029	0.129
$\bar{\omega }$	—	—	−0.117	0.250	−0.104	0.137	—	—	−0.079	0.142	−0.038	0.076
Var(ω)	—	—	−0.012	0.095	−0.010	0.052	—	—	−0.031	0.083	−0.008	0.030
MDP	0.042	0.089	—	—	—	—	0.015	0.056	—	—	—	—
**Test length = 40**												
Discrimination	0.028	0.072	—	—	—	—	0.008	0.046	—	—	—	—
Location	0.000	0.068	0.000	0.077	0.000	0.125	0.000	0.049	0.000	0.053	0.000	0.089
Threshold	0.000	0.111	—	—	—	—	0.000	0.076	—	—	—	—
$\bar{\theta }$	—	—	0.025	0.077	−0.006	0.067	—	—	−0.005	0.041	−0.007	0.058
Var(θ)	—	—	−0.035	0.147	−0.053	0.139	—	—	−0.005	0.094	−0.047	0.102
$\bar{\omega }$	—	—	−0.065	0.148	−0.085	0.113	—	—	−0.052	0.108	−0.050	0.074
Var(ω)	—	—	−0.003	0.066	−0.009	0.027	—	—	−0.021	0.063	−0.001	0.023
MDP	0.052	0.090	—	—	—	—	0.014	0.056	—	—	—	—

*—, not applicable because of model constraints*.

Next, the person parameter recovery was examined by inspecting the classification accuracy and RMSE values of the random-effect parameter estimates ($\hat{\theta }$ and $\hat{\omega }$), which are presented in Table 5. These criteria were averaged over 30 replications. The long test length and large sample size were associated with a more precise estimation for both the θ latent trait and ω weight parameter and with a higher correct classification rate. The superior parameter estimation was more significant for longer test lengths than larger sample sizes. The θ target latent trait was recovered more accurately than the ω weight parameter for the examinees. When the response categories increased from four to six, the RMSE values substantially decreased and the correct classification rates increased. In addition, the mixture ERS-GPCM-CD yielded slightly better person parameter recovery than the mixture ERS-GPCM.

**Table 5:** **Statistical summary of the person parameter recovery in the mixture ERS-IRT models**.

**Model**	**Mixture ERS-GPCM**								**Mixture ERS-GPCM-CD**							
**Point**	**4**				**6**				**4**				**6**			
**Sample size**	**1000**		**2000**		**1000**		**2000**		**1000**		**2000**		**1000**		**2000**	
**Test length**	**20**	**40**	**20**	**40**	**20**	**40**	**20**	**40**	**20**	**40**	**20**	**40**	**20**	**40**	**20**	**40**
**CRITERION**																
Mean RMSE(θ)	0.306	0.232	0.304	0.227	0.250	0.186	0.247	0.183	0.303	0.231	0.299	0.227	0.246	0.187	0.241	0.181
Mean RMSE(ω)	0.663	0.451	0.607	0.418	0.488	0.335	0.455	0.315	0.610	0.411	0.557	0.373	0.454	0.326	0.421	0.296
Mean CCR	0.744	0.883	0.766	0.886	0.866	0.951	0.878	0.954	0.756	0.894	0.788	0.897	0.877	0.957	0.883	0.959

*CCR, correct classification rate*.

Compared to the item parameter recovery in Tables 1–4, the person parameters appeared to have been recovered poorly because of their slightly larger RMSE values. This result was not surprising because the precision of latent trait estimation largely depends on the amount of information that items provide, and using a sufficient number of test items with maximum information for each respondent can improve the quality of person parameter estimation (Embretson and Reise, 2000). A fixed-length test was used in this study, so we did not expect that the precision of the latent trait estimation could reach an excellent level for all respondents, such as in computerized adaptive testing (CAT). Nevertheless, our results were comparable to those of previous studies relative to mixture IRT modeling when similar sample sizes and test lengths were used (e.g., Huang, 2016).

### Consequences of ignoring mixtures of latent classes in response styles

The consequences of ignoring mixtures of latent classes in response styles were demonstrated by fitting the ERS-GPCM to the mixture ERS-GPCM data when data were generated from the responses of 2000 examinees to 40 four-point items. We focused on the person parameter recovery, and a total of 30 replications were conducted. When the ω weight parameter was estimated with the ERS-GPCM (mixtures ignored), statistical hypothesis testing was conducted to examine whether the estimate was significantly different from one. With a nominal α of 0.05, the examinees were grouped into the ERS class if their statistics were lower than −1.96 and into the MRS class if their statistics were higher than 1.96. The examinees with statistics that were not significantly different from one were identified as within the normal class (see Jin and Wang, 2014a).

**Figure 1:**
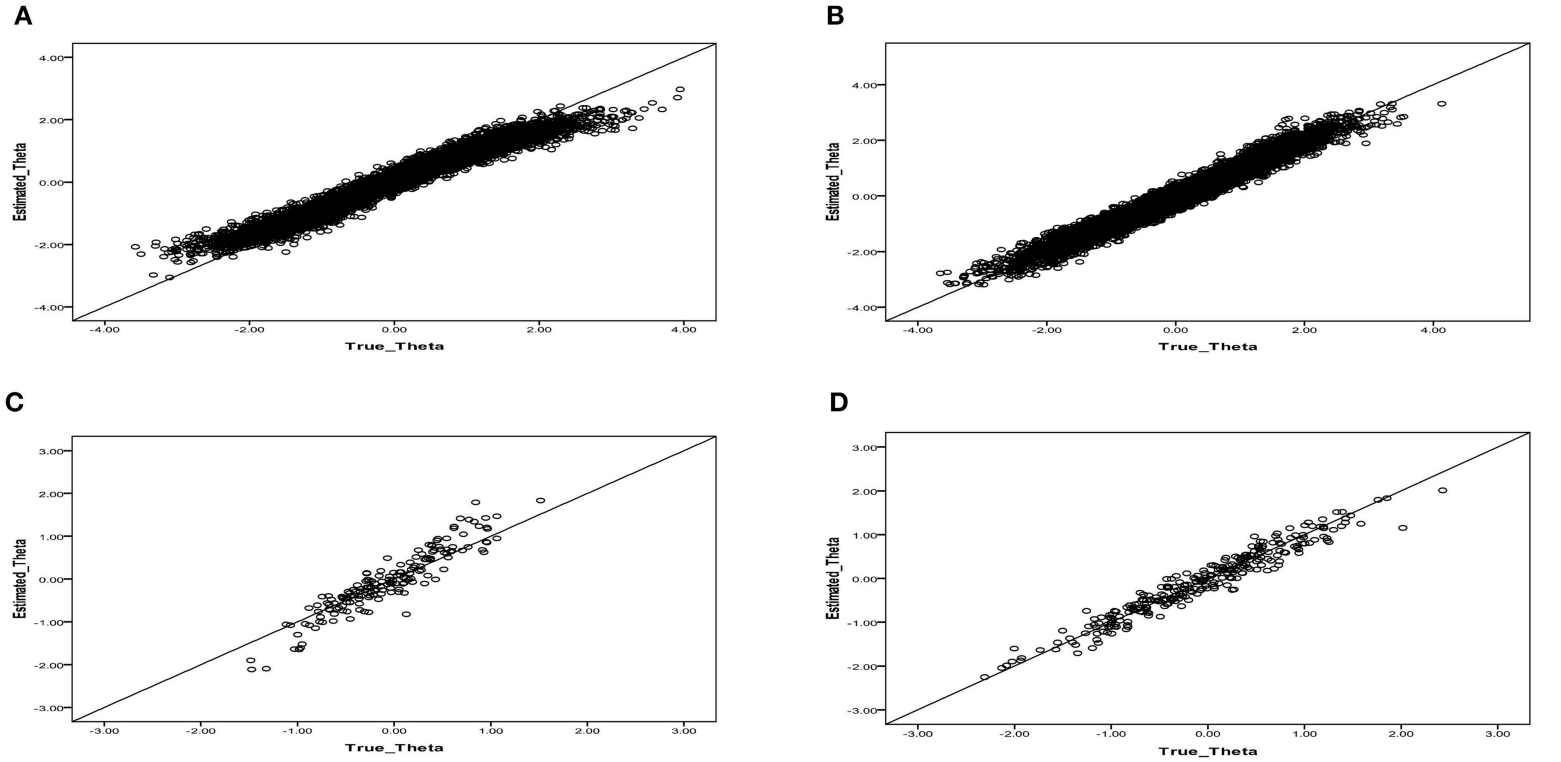
**Relationship between the true and estimated latent trait parameters when respondents were misclassified by using the single-class ERS-GPCM to fit the mixture ERS-GPCM data. (A)** Individuals in the MRS class that were misclassified into the normal class. **(B)** Individuals in the ERS class that were misclassified into the normal class. **(C)** Individuals in the normal class that were misclassified into the MRS class. **(D)** Individuals in the normal class that were misclassified into the ERS class.

The results showed that the mean correct classification rate decreased from 0.886 (with mixtures) to 0.687 (without mixtures) and that the mean RMSE values had higher values of 0.235 and 0.542 for the θ and ω parameter estimates, respectively, compared to the lower RMSE values in the mixture ERS-GPCM. We investigated the effect of misclassification on the latent trait estimation of respondents and observed whether any systematic influences on which respondents were misclassified existed by inspecting the relationship between the true and estimated latent traits for respondents who were misclassified into the normal, ERS, or MRS classes. When the respondents of the MRS class were misclassified into the normal class, as shown in Figure 1A, the latent trait estimates for the respondents with high ability levels were somewhat underestimated and those with low ability levels were somewhat overestimated. Identical patterns were found when the respondents of the ERS class were misclassified into the normal class (see Figure 1B). The number of misclassifications from the normal to MRS or ERS class, as shown in Figures 1C,D, respectively, was lower, and the patterns appeared to be less systematic compared to the above misclassifications. Few respondents (less than six respondents) were incorrectly classified as within the ERS (or MRS) class when their true class was MRS (or ERS), so these plots are not presented here.

The over- and under-estimation of latent traits on the extreme end of a continuum trait scale may not be associated with the levels of ERS or MRS that respondents truly possessed because the resulting classification was based on the ω parameter and its corresponding SE estimation and because the ω parameter was assumed to be independent of the θ parameter. One plausible reason for this phenomenon was that the EAP estimates for θ tended to shrink to the zero mean of the θ prior distribution when a finite number of items was present (Wainer and Thissen, 1987). In addition, the number of ERS and MRS respondents who were misclassified into the normal class was found to be larger than those who were misclassified into the ERS and MRS classes when their true class was normal, possibly because the statistical hypothesis testing for the ERS and MRS classes was too conservative to accurately identify their true latent classes.

Although, not shown in detail, the same findings applied to other conditions of different sample sizes, test lengths, and option numbers. In summary, ignoring the mixtures of latent classes by fitting the ERS-GPCM to the mixture ERS-GPCM data yielded poor parameter estimation, which suggests that the response patterns of different response styles should be considered in data analysis and should have a large effect on person parameter recovery.

### Empirical study

Wang (2008) surveyed college students' performances on emotional intelligence by using the scale of “Chinese Emotional Intelligence,” which consists of three subscales of emotion management (9 items), emotion awareness (16 items), and interpersonal interaction (19 items). A total of 2363 students in Taiwan were recruited to respond to 44 five-point items (1 = *strongly unconfident*, 2 = *slightly unconfident*, 3 = *neutral*, 4 = *slightly confident*, and 5 = *strongly confident*). A higher score indicates a greater degree of confidence on the performance of emotional intelligence, and a partial credit model was used to fit the data. Variations in the mixture ERS-GPCM and mixture ERS-GPCM-CD, which considered unidimensional/multidimensional approaches and the number of latent classes (one, two, and three), were developed to simultaneously evaluate the multidimensional nature and latent classes of response styles. Twelve extension models (2 types of mixture ERS-GPCMs × 2 types of dimensionality × 3 types of latent classes) were used to fit the data. Different models were compared by using the Bayesian Information Criterion (BIC; Schwarz, 1978), which is commonly used in mixture IRT models with Bayesian estimation (e.g., Cho and Cohen, 2010; Jin and Wang, 2014b). A smaller BIC value indicates a good model-data fit.

The model comparison results are listed in Table 6, in which the mixture ERS-GPCM-CD with three latent classes was selected as the best-fitting model because of its smallest BIC value of 192,100. Among the three latent classes, the first class was constrained as a normal class, the second class was identified as the ERS class with an estimated mean ω of −0.5 and variance of 0.05 in a lognormal distribution, and the third class was classified as the MRS class with an estimated mean ω of 0.4 and variance of 0.02 in a lognormal distribution. In addition, the ERS and MRS classes had a higher estimated mean θ than the normal class for the three subscales. The θ variance was estimated as 1.14, 0.95, and 0.63 for the three respective scales, and the covariance was estimated as 0.72 between the emotion management scale and the emotional awareness scale, 0.63 between the emotion management scale and the interpersonal interaction scale, and 0.59 between the emotion management scale and the interpersonal interaction scale, which suggests that the correlations between the three dimensions were substantial enough to consider.

**Table 6:** **BIC values for the 12 competing models**.

	**Number of latent classes**		
**Model**	**1**	**2**	**3**
**UNIDIMENSIONAL MODEL**			
Mixture ERS-GPCM	210,600	208,500	202,900
Mixture ERS-GPCM-CD	210,400	208,400	202,900
**MULTIDIMENSIONAL MODEL**			
Mixture ERS-GPCM	198,500	197,200	193,900
Mixture ERS-GPCM-CD	198,500	196,100	192,100

The developed models can estimate different sets of item difficulty across latent classes, so differences in the item difficulty parameter estimates between the three classes were calculated as absolute values. The magnitudes of the difficulty differences ranged from 0.02 to 1.41 (*mean* = 0.64) between the normal and ERS classes and from 0.04 to 1.65 (*mean* = 0.55) between the normal and MRS classes, which suggest that the differences in item difficulty were not trivial and that different response styles contributed to different response patterns. The discrimination parameters with respect to the θ latent trait were estimated between 0.59 and 2.37 (*mean* = 1.55), and the three MDP estimates were 3.40, 3.84, and 3.76 for the three subscales. As argued earlier, one advantage of the ERS-GPCM-CD in analyzing response styles is its capacity to represent the propensity of an item to elicit extreme responses. The item's discriminating power on the ω parameter can be obtained according to Equation (7) by combining the estimated discrimination parameters with the MDP estimates. For illustration purposes, the two items with the highest and lowest discrimination parameters are demonstrated. One of the items in the emotion management subscale is “I am quite capable of presenting my negative emotions to others,” which had the highest discrimination parameter estimate. An item that measured the emotion awareness dimension was “I am aware of the non-verbal messages other people send,” which had the lowest likelihood of eliciting ERS or MRS relative to other items. Because these items were translated from the Chinese version, the item characteristics and attributes (e.g., word numbers or clarity) that may contribute to ERS performance should be further investigated by domain and testing experts.

Finally, the latent trait estimates that were obtained from three different models, which were the mixture ERS-GPCM-CD (i.e., the best-fitting model), the ERS-GPCM-CD (no mixtures), and the GPCM (without incorporating ERS), were compared to investigate the practical effect of ignoring the mixtures and ERS on latent trait estimation. We ranked these estimates in order and calculated the rank order changes in the absolute values between the latent trait estimates that were obtained from the gold standard and the other two parsimonious models by treating the latent trait estimates of the mixture ERS-GPCM-CD as the gold standard. A large rank-order change indicated that the practical effect could not be neglected. The maximum rank-order changes were 513 (*mean* = 90), 380 (*mean* = 82), and 414 (*mean* = 74) for the three subscales, which measured emotion management, emotion awareness, and interpersonal interaction, respectively, when comparing the gold standard to the single-class ERS-GPCM-CD. When comparing the gold standard to the traditional GPCM, the maximum rank-order changes substantially increased to 1162 (*mean* = 191), 1079 (*mean* = 185), and 1151 (*mean* = 180) for the three respective subscales. Although, the results showed that the effect of ignoring ERS on individuals' latent trait estimation was more severe than that when ignoring the mixtures of latent classes, both effects were non-trivial because individuals with a high degree of EQ were sometimes incorrectly identified as exhibiting a low level of EQ and vice versa. In addition, the correlation of the ω weight parameter estimate that was obtained from the gold standard and the single-class ERS-GPCM-CD was 0.94, which suggests that the ω weight parameter appeared to be less influenced by the neglect of mixtures in the latent distributions among respondents.

In summary, different response patterns and mixtures of latent classes should be considered in this example when diverse response styles are presented, and the mixture ERS-GPCM-CD that was proposed in this study can model the response tendencies of respondents toward middle or extreme options, thus distinguishing mixtures of latent classes, with each having different sets of item parameters, and providing a clear indication to evaluate the likelihood that an item elicits an ERS or MRS.

## Conclusions and discussion

The phenomenon of respondents' extreme responses to rating scale items in attitude and personality assessment distorts the measurement validity and leads to measurement inequivalence or DIF (e.g., Bolt and Johnson, 2009; Morren et al., 2012). Several approaches have been proposed to control for the influence of ERS (or the opposite effect, MRS) on item responses, and each has applicability and practicability limitations in that respondents' tendencies toward ERS cannot be jointly quantified and classified, and the role that ERS play in the creation of DIF is not clear. Latent DIF may coincide with ERS, so we developed a new class of mixture IRT models in this study to simultaneously identify latent classes with respect to different response styles (i.e., normal, ERS, and MRS) and to detect possible latent DIF among these classes. A distinguishing characteristic of the extended models comes from the use of ordered-category IRT models to account for the influence of ERS, the measurement of the tendencies of ERS on a continuum scale, the classification of different response styles in both target (θ) and ERS (ω) dimensions, and the assessment of whether DIF items are flagged because of different levels of ERS tendency.

As indicated by the simulation results, a long test and large sample can improve parameter estimation in the new class of mixture ERS-IRT models, and the precision of the parameter estimates increases with the number of response options. Imposing a distinct set of discrimination parameters on the ω weight parameter demonstrated the advantages of evaluating whether test items are designed appropriately to suppress the tendency toward ERS or MRS among examinees. The model parameters could be recovered better than the person parameters, although both types of parameters were estimated satisfactorily. When mixtures of latent classes that resulted from different response styles occurred and a different set of item parameters was suspected among these latent classes, the use of non-mixture ERS-IRT models to fit the data results in biased parameter estimation and reduced the correct classification rates with respect to the response styles. The latent trait parameters for the misclassified respondents with high or low ability appeared to be underestimated or overestimated because the EAP estimator caused the trait level estimates to regress toward the zero mean of the θ prior distribution.

The real data analysis showed that the mixture ERS-GPCM-CD fit better than the other ERS-GPCMs, and the MDP could be used to evaluate the characteristics that an item has to elicit ERS or MRS tendencies. Items that were identified as having a greater likelihood to elicit ERS or MRS tendencies may have come from the use of negative words to describe emotion management because similar phenomena to that item were observed for other items with higher discrimination parameter estimates with respect to the ω weight parameter. The consequences of ignoring the mixtures of latent classes and the effect of ERS resulted in larger rank-order changes for the three subscales compared to the gold standard (i.e., the mixture ERS-GPCM-CD). Comparing the ω weight parameter estimates that were obtained from the gold standard with those that were obtained from the single-class ERS-GPCM indicated that the effect of ignoring the mixtures was not substantial because the single-class ERS-GPCM did not calibrate a separate set of item difficulty parameters for the three latent classes and because the ω weight parameter was directly associated with the threshold parameters rather than the item difficulty parameters.

Although, the application of the developed models to real data analysis was demonstrated in the context of multidimensionality, the results were expected to remain valid because the ERS-IRT models can be directly applied to the analysis of a multidimensional test (Jin and Wang, 2014a). In addition, multiple-scale measurements can provide more precise estimations for the intended-to-be-measured and ERS dimensions than a unique-scale measurement (Bolt and Newton, 2011).

As one of the reviewers reminded, a small number of test items may be administered to a limited number of subjects during real testing situations, and the effectiveness of the proposed model in such situations should be evaluated. We attempted to decrease the number of test items and respondents (e.g., 10 or 15 items and 200 or 500 respondents) in a new simulation but found that the chains in WinBUGS for the combinations of different conditions failed to converge stochastically. In the framework of mixture IRT modeling, a sufficient number of items are required to correctly identify latent classes of respondents that have homogeneous item response patterns, and the number of respondents in each latent class must be sufficient enough to provide precise random-effect variance estimations. The most common simulation settings in previous studies that were relevant to the mixture Rasch model were the use of more than 25 items and more than 500 examinees (e.g., Meyer, 2010; Dai, 2013; Choi and Wilson, 2015). When a multi-parameter mixture IRT model was used, the number of examinees and items had to be increased substantially because of its complex nature (e.g., Cho et al., 2014; Huang, 2016). Our developed models belong to the family of multi-parameter IRT models and must identify three different latent classes, so the use of extremely small numbers of individuals and test items is not practical and therefore not recommended.

An additional simulation was conducted to further investigate the minimum number of individuals and items that are required by the proposed mixture ERS-IRT models, in which the responses of 500 individuals to 20 four-point items were generated by using the mixture ERS-GPCM and the other settings were identical to those in the first simulation study. Several data sets were found to fail during model parameter convergence, so only the results in which the chains became stationary were reported. Compared to the results of 1000 individuals' responses to 20 items, which are summarized in Tables 1,5, all the bias values were very different from zero and the mean RMSE values were 0.137, 0.219, 0.256, 0.161, 0.314, 0.359, and 0.345 for the discrimination parameter estimates, the location parameter estimates (across classes), the threshold parameter estimates, the θ mean estimate (across classes), the θ variance estimate (across classes), the ω mean estimate (across classes), and the ω variance estimate (across classes), respectively. With respect to the person parameter recovery, the RMSE values increased to 0.313 and 1.871 for the θ and ω parameter estimates, respectively, and the correct classification rate decreased to 0.707. Although most of the model and person parameter recoveries appeared to be marginally acceptable during this simulation, we recommend that a sufficient number of individuals (i.e., at least 1000 subjects) and an adequate number of items (i.e., at least 20 items) should be used to maximize the benefits of our proposed models when the ERS phenomenon and different response patterns are suspected among respondents because of the uncertainty in model parameter convergence with Bayesian estimation and the poor estimation for ω.

Recently, Plieninger (2016) conducted simulations to assess the consequences of ignoring response styles (including ERS) and concluded that response styles hardly bias the results based on self-report data when the latent trait(s) and the response style are uncorrelated. His findings seemingly contrast our conclusions that different tendencies toward ERS that are possessed by subjects should not be ignored and that the effects of ERS on the measurement outcomes are not negligible. We address several differences between our and his studies and comment on the controversial issues as follows. First, the ERS options in the multidimensional Rasch model for ERS that was proposed by Plieninger should be determined arbitrarily by researchers in advance, and different categorizations of extreme responses will lead to different results (Jin and Wang, 2014a), similar to the MNRM for ERS (Bolt and Johnson, 2009; Johnson and Bolt, 2010; Bolt and Newton, 2011). When the response categories increased, determining which options truly represented ERS (e.g., eight-point rating scale) became more difficult. Second, classical reliability and validity indices were used to evaluate the degree of bias from ERS by using the raw scores of respondents. Such an analysis may distort the effects of ERS on scale measures when ignoring the ERS's effect. The literature states that the use of raw scores and IRT trait levels can lead to different statistical conclusions; for example, spurious interactions can occur by using the raw scores when no interaction exists in the true trait levels (Embretson, 1997; Embretson and Reise, 2000). Third, how well Plieninger's model works when the discrimination parameters must be estimated is uncertain. Accordingly, we reserve the present conclusions.

The simulations were performed on personal computers with a 3.5-GHz Intel Core I7. Each replication took approximately several days to 1 week, and the MCMC simulation time increased dramatically when multiple MCMC chains were involved. This computation time was not feasible or efficient for comprehensive simulations, so only 30 replications were conducted in this study. In fact, we attempted to increase the number of replications for several simulation conditions and found that the sampling variation across replications was very small. Additionally, a small to moderate number of simulation replications were used in previous studies relative to the development and extension of mixture IRT models when using WinBUGS; for example, five replications were used in the study by Cho and Cohen (2010), 10 replications by Dai (2013), and 30 replications by Choi and Wilson (2015). Twenty replications were used in the study by Jin and Wang (2014a) for the development of ERS-IRT models with the use of WinBUGS.

The availability of a user-friendly computer program that can be used to fit the proposed models to data is very important for practical applications. To the best of our knowledge, no commercial computer software, such as the Mplus computer program, is ready to analyze response data with mixture ERS-IRT models, which may limit the applications of the developed models to real testing situations. Nevertheless, the basic WinBUGS commands for the proposed models are readily available upon request, so ordinary users do not have to struggle with the derivation of parameter estimation procedures and can modify a few lines of previously written code for their customized models.

Two practical questions may be addressed by researchers when using the proposed mixture ERS-IRT models to fit data in real testing situations. First, should researchers routinely apply these new models to analyze data to detect ERS? The answer depends on the research purposes and the availability of prior information for respondents. If the results of data analysis are used for cross-culture comparison or individual diagnostic intentions, the test validity and score inference are very important and the effect of ERS should be examined and controlled. Furthermore, some psychological traits (e.g., trait anxiety) or demographic variables (e.g., educational level) have been found to be correlated to ERS (Paulhus, 1991; Meisenberg and Williams, 2008; Bolt and Johnson, 2009; De Beuckelaer et al., 2010; Van Rosmalen et al., 2010). If researchers can obtain such additional information for the respondents before analyzing the data (for example, most respondents have low educational level), researchers should use a mixture ERS-IRT model to fit the data to control for ERS.

The second question is a more statistical issue: how many latent classes should be hypothesized by researchers during analysis when using mixture ERS-IRT models? This question can be answered by the demonstration of the empirical analysis. When ERS is suspected in the data responses, the ω weight parameter is added into polytomous IRT models to quantify the ERS tendency, and three latent classes (i.e., the normal, ERS, and MRS classes) can be expected if different response patterns among the three latent classes are observed. However, ideal classifications may not be present if the number of respondents in certain latent classes is extremely small or if the response patterns between classes are unapparent. As in traditional mixture IRT models (e.g., Wilson, 1989), the developed mixture ERS-IRT models simultaneously consider theoretical and statistical approaches. The three plausible latent classes can be hypothesized and compared from a theoretical perspective. Parsimonious mixture ERS-IRT models with one or two class(es) may provide better fits to the data after conducting model comparison from a statistical perspective. More than three latent classes may arise during data analysis, but this phenomenon is beyond the scope that the proposed model can interpret.

There are several directions for future studies. First, longitudinal surveys of attitude or personality assessment are common in large-scale measurements. Previous studies indicated that the tendency of ERS is highly internally consistent over time and is considered a stable trait (Berg, 1953; Hamilton, 1968; Bachman and O'Malley, 1984; Weijters et al., 2010), but some exceptions have been described (Meisenberg and Williams, 2008). The incorporation of multilevel IRT models (Huang, 2015) with mixture ERS-IRT models to form multilevel mixture ERS-IRT models may serve as a possible solution to this issue. Second, specific item features may elicit ERS tendencies, and the probability of endorsing each option on rating-scale items can be formulated as a function of the item features. Thus, mixture ERS-IRT models can accommodate the linear logistic test model (Fischer, 1995) to simultaneously consider both respondents' ERS tendencies and item characteristics. Finally, as mentioned above, sequential IRT models have been applied to distinguish the target latent traits and the ERS tendencies of respondents (e.g., Böckenholt, 2012; Thissen-Roe and Thissen, 2013). How these approaches would work for the identification of different latent classes of response styles and the simultaneous detection of latent DIF among items, which can be compared to our mixture ERS-IRT models in terms of estimation efficiency, deserves further investigation.

## Author contributions

The author confirms being the sole contributor of this work and approved it for publication.

## Funding

This study was partly supported by the Ministry of Science and Technology, Taiwan (Grant NO. 104-2410-H-845 -002 -MY2).

### Conflict of interest statement

The author declares that the research was conducted in the absence of any commercial or financial relationships that could be construed as a potential conflict of interest.
